# Comparison of Emergency Department Disposition Times in Adult Level I and Level II Trauma Centers

**DOI:** 10.5811/westjem.20523

**Published:** 2024-10-22

**Authors:** Sierra Lane, Jeffry Nahmias, Michael Lekawa, John Christian Fox, Carrie Chandwani, Shahram Lotfipour, Areg Grigorian

**Affiliations:** * University of California, Irvine, Department of Surgery, Division of Trauma, Burns and Surgical Critical Care, Orange, California; † University of California, Irvine, Department of Emergency Medicine, Orange, California

## Abstract

**Introduction:**

The efficient utilization of resources is a crucial aspect of healthcare, particularly in both Level I and Level II American College of Surgeons (ACS)-verified trauma centers. The effect of resource allocation on emergency department length of stay (ED-LOS) of trauma patients has remained under-investigated. As ED crowding has become more prevalent, especially at quaternary care centers, an evaluation of the potential disparities in ED-LOS between Level I and Level II trauma centers is warranted. We hypothesized a longer ED-LOS at Level I centers compared to Level II centers.

**Methods:**

We queried the 2017–2021 Trauma Quality Improvement Process (TQIP) database for trauma patients ≥18 years of age presenting to either a Level-I or -II center. The TQIP defines ED-LOS as the time from arrival until the time an ED disposition (admission or discharge) order is written. We excluded transferred patients and those with missing data regarding ACS trauma center verification level. We performed bivariate analyses, as well as subgroup analyses based on location of disposition.

**Results:**

Of 2,225,067 trauma patients, 59.3% (1,318,497) received treatment at Level I centers. No significant differences were found in Injury Severity Scores between patients admitted to the operating room or non-intensive care unit (ICU) locations, or discharged home from Level-I and -II centers (all *P* < 0.05). The ED-LOS for trauma patients was longer at Level-I centers for all patient categories: overall (198 vs 145 minutes [min], *P* < 0.001), discharged home (286 vs 160 min, *P* < 0.001), non-ICU admissions (234 vs 164 min, *P* < 0.001), and those requiring surgery (126 vs 101 min, *P* < 0.001).

**Conclusion:**

Even when treating patients with similar injury severity, trauma patients at Level I trauma centers had longer ED-LOS compared to Level II centers, irrespective of the patients’ final disposition (surgery, non-ICU admission, or discharge). To optimize resource utilization and alleviate ED saturation, further research must delve into the underlying causes of these discrepancies to identify best practices and solutions.

Population Health Research Capsule What do we already know about this issue?

*Prolonged ED length of stay (LOS) is linked to adverse clinical outcomes and highlights the importance of rapid and well-coordinated emergency care.*

What was the research question?

*How does ED-LOS for trauma patients differ between Level I and Level II trauma centers?*

What was the major finding of the study?

*The ED-LOS for trauma patients at Level I centers was longer overall (198 vs 145 minutes, P < 0.001) compared to Level II centers.*

How does this improve population health?

*This research enhances our understanding of patient experience and quality of healthcare by addressing ED crowding, a longstanding issue nationwide.*

## INTRODUCTION

Trauma continues to pose a significant public health challenge that places substantial demands on healthcare systems. Since 2010, trauma has consistently been the leading cause of death for young adults.^1^ The Coalition for National Trauma Research reports that trauma accounts for approximately 41 million emergency department (ED) visits each year as well as two million hospital admissions annually.^2^ In this context, the length of stay (LOS) in the ED acts as a key metric, reflecting the efficiency and effectiveness of patient care. Prolonged ED-LOS is associated with adverse clinical outcomes, including increased risk of hospital-acquired infections, delays in the administration of critical medications, and increased mortality, which highlights the importance of rapid and well-coordinated emergency care.^3^ ^–^ ^10^ Existing literature highlights disparities in ED-LOS across various medical centers; however, there is a significant lack of data focusing on trauma centers.^11^

Trauma centers are designated by the American College of Surgeons (ACS) based on patient volume, staffing, resources, injury prevention, and education.^12^ This tiered structure has enabled a shift from traditional, hospital-centric models to a more integrated, regionalized system of trauma care.^13^ Despite existing studies highlighting the complexities of trauma cases and the impact of prolonged ED-LOS, there remains a substantial gap in research concerning how resource allocation affects ED-LOS for trauma patients, particularly between various levels of trauma centers.^14^ ^–^ ^18^ These levels may differ in terms of resources and capabilities in the ED, with Level I trauma centers (L1TC) typically handling more complex cases and having more comprehensive resources compared to Level II trauma centers (L2TC).

The importance of investigating ED-LOS differences between L1TCs and L2TCs has become more pronounced in the wake of the COVID-19 pandemic. The pandemic affected trauma mechanisms and outcomes including exacerbated ED crowding, a longstanding issue in healthcare, and posed unique challenges to trauma care, particularly in higher level trauma centers, which often serve as quaternary care facilities.^19^ ^–^ ^24^ Crowding leads to delays in care and a bidirectional impact on both trauma and non-trauma patients. The influx of trauma patients to the ED reallocates staff and resources from other patients undergoing simultaneous evaluation and treatment, increasing their ED-LOS.^25^ ^,^ ^26^

In this study we aimed to analyze a large United States trauma database to compare ED-LOS between adult trauma patients at L1TCs and L2TCs. We hypothesized an increased ED-LOS at L1TCs compared to L2TCs. This research may help improve patient experience and quality of healthcare as ED crowding continues to impact hospitals nationwide.

## METHODS

This study was deemed exempt from institutional board review, and a waiver of consent was granted for use of a de-identified national database. We performed a retrospective analysis of the Trauma Quality Improvement Program (TQIP) database from 2017–2021. Patients ≥18 years of age presenting to either an ACS-verified L1TC or L2TC were included. We excluded all patients transferred from another facility as well as those with missing data regarding ACS trauma center-verification level. Our primary focus was to accurately assess ED-LOS for trauma patients. Including transfer patients would have introduced confounding factors that could have significantly skewed our analysis. Trauma transfer patients may have already undergone extensive evaluations and imaging at the initial hospital, which can artificially shorten their ED-LOS at the receiving hospital. Additionally, some of these patients may have been pre-accepted by the trauma team, resulting in a more expedited admission process compared to non-transfer patients. Therefore, including transfer patients would not provide an accurate representation of ED-LOS for trauma patients. We compared two groups: adult trauma patients treated at L1TCs vs L2TCs. This included a comparison of all patients regardless of level of care.

We collected patient demographic variables including age and prehospital comorbidities such as diabetes mellitus, hypertension, anticoagulant therapy, mental or personality disorder, smoking status, houselessness, and substance use. The injury profile included the Injury Severity Score (ISS), and the Abbreviated Injury Scale (AIS) of the head, abdomen, and thorax. We also collected vitals on arrival including hypotension (systolic blood pressure ≤ 90 millimeters of mercury), tachycardia (heart rate > 120 beats per minute), and tachypnea (respiratory rate > 22 breaths per minute). The primary outcome measured was ED-LOS. Additionally, we collected patient disposition from the ED, including admission to the general hospital floor, intensive care unit (ICU), operating room (OR), or discharge to home. We also analyzed inpatient complications, such as acute kidney injury, cardiac arrest, unplanned intubation, ventilator-acquired pneumonia, and deep vein thrombosis. We contrasted patient characteristics, injury profiles, complications, and dispositions between adult patients treated at L1TCs and L2TCs.

We performed bivariate analyses using a Mann-Whitney U test to compare continuous variables and chi-square to compare categorical variables. We report categorical data as percentages and continuous data as medians with interquartile range (IQR) or as means with standard deviation. A multivariable logistic regression analysis was also performed to determine the associated risk of mortality and complications. Each model included known risk factors for mortality and inhospital complications for trauma patients including age, vitals on admission, mechanism, ISS, and the presence of traumatic brain injury.^27^ ^–^ ^30^ These covariates were determined by co-author consensus and review of the literature. All *P*-values were two-sided with a statistical significance level of <0.05. We performed all analyses with SPSS Statistics for Windows v29 (IBM Corp, Armonk, NY). The Strengthening the Reporting of Observational Studies in Epidemiology (STROBE) checklist was used to ensure adherence with established guidelines for reporting observational studies.^31^

## RESULTS

### Demographics, Characteristics, and Injuries of Patients at Level-I vs II-Trauma Centers

Of 2,225,067 patients, 59.3% (1,318,497) received treatment at a L1TC and 40.7% (906,570) at a L2TC. Patients at L1TCs were generally younger (median 50 vs 58 years, *P* < 0.001) than at L2TCs. The L1TC and L2TC patients had a similar median ISS of 9. However, patients at L2TCs had higher rates of the following prehospital comorbidities: anticoagulant therapy (12.1% vs 8.8%, *P* < 0.001); diabetes mellitus (15.2% vs 13.5%, *P* < 0.001), and hypertension (37.2% vs 31.9%, *P* < 0.001). Patients at L1TCs were more often houseless (1.6% vs 1.2%, *P* < 0.001); more often underwent blood transfusions compared to L2TCs (5.9% vs 3.6%, *P* < 0.001), and had higher rates of substance use disorder (9.0% vs 6.3%, *P* < 0.001) (Table 1). Patients treated at L1TCs also had increased rates of high-grade injuries (AIS > 3) to the head (14.4% vs 14.2%, *P* < 0.002), abdomen (4.8% vs. 3.5%, *P* < 0.001), and thorax (15.8% vs. 13.1%, *P* < 0.001) (Table 2).

**Table 1.** Demographics, comorbidities, and vital signs of adult trauma patients treated at level I vs level II trauma centers.

Characteristic	Level I (n = 1,318,497)	Level II (n = 906,570)	*P*-value
Age, year, median (IQR)	50 (32, 68)	58 (39, 77)	<0.001
Comorbidities, n (%)			
Alcohol use disorder	92,090 (7.1%)	54,266 (6.1%)	<0.001
Houselessness^*^	4,834 (1.6%)	2,383 (1.2%)	<0.001
Congestive heart failure	48,149 (3.7%)	40,712 (4.5%)	<0.001
Current smoker	306,022 (23.5%)	168,447 (18.8%)	<0.001
Chronic renal failure	20,982 (1.6%)	15,992 (1.8%)	<0.001
Cerebrovascular accident	32,748 (2.5%)	25,637 (2.9%)	<0.001
Diabetes mellitus	175,825 (13.5%)	135,779 (15.2%)	<0.001
Hypertension	416,425 (31.9%)	334,025 (37.2%)	<0.001
Chronic obstructive pulmonary disease	76,547 (5.9%)	65,353 (7.3%)	<0.001
Cirrhosis	15,868 (1.2%)	9,301 (1.0%)	<0.001
Dementia	53,592 (4.1%)	53,164 (5.9%)	<0.001
Anticoagulant therapy	114,428 (8.8%)	108,082 (12.1%)	<0.001
Angina pectoris	1,872 (0.1%)	2,137 (0.2%)	<0.001
Myocardial infarction	7,609 (0.6%)	5,833 (0.7%)	<0.001
Peripheral arterial disease	10,938 (0.8%)	9,362 (1.0%)	<0.001
Substance use disorder	117,680 (9.0%)	56,160 (6.3%)	<0.001
Vitals on admission, n (%)			
Hypotension (SBP < 90)	59,051 (4.6%)	29,024 (3.3%)	<0.001
Tachycardia (HR > 120)	99,388 (7.7%)	55,816 (6.3%)	<0.001
Tachypnea (RR > 22)	223,774 (17.6%)	136,745 (15.5%)	<0.001
Blood transfusion, n (%)	78,273 (5.9%)	32,230 (3.6%)	<0.001

* Only includes 2021 data.

*IQR*; interquartile range; *HR*, heart rate; *RR*, respiratory rate; *SBP*, systolic blood pressure.

**Table 2.** Injuries for adult trauma patients treated at level I vs level II trauma center.

Characteristic, n (%)	Level I (n = 1,318,497)	Level II (n = 906,570)	*P*-value
ISS, median (IQR)	9 (4.5, 13.5)	9 (6,12)	<0.001
Blunt mechanism, n (%)	1,107,121 (84.0%)	810,732 (89.4%)	<0.001
AIS grade > 3, n (%)			
Head	189,248 (14.4%)	128,778 (14.2%)	<0.002
Abdomen	63,011 (4.8%)	31,659 (3.5%)	<0.001
Thorax	208,098 (15.8%)	118,421 (13.1%)	<0.001
Injury, n (%)			
Brain	221,032 (16.8%)	134,573 (14.8%)	<0.001
Liver	43,616 (3.3%)	18,070 (2.0%)	<0.001
Small intestine	17,861 (1.4 %)	6,904 (0.8 %)	<0.001
Colon	16,162 (1.2%)	5,979 (0.7%)	<0.001
Rectum	2,072 (0.2%)	675 (0.1%)	<0.001
Kidney	20,043 (1.5%)	9,370 (1.0%)	<0.001
Spleen	36,477 (2.8%)	17,827 (2.0%)	<0.001
Pancreas	4,756 (0.4%)	1,687 (0.2%)	<0.001
Stomach	5,138 (0.4%)	1,723 (0.2%)	<0.001

*AIS*, Abbreviated Injury Scale; *IQR*, interquartile range; *ISS*, Injury Severity Scale.

### ED-LOS of L1TC and L2TC

Patients at L1TCs were admitted at higher rates to the ICU (19.3% vs 17.6%, *P* < 0.001) and directly to the OR (13.7% vs 10.6%, *P* < 0.001), while patients at L2TCs were admitted at higher rates to the general hospital floor/ward (57.1% vs 55.4%, *P* < 0.001) and discharged home (9.8% vs 7.9%, *P* < 0.001). The L1TC patients had increased median ED-LOS for all dispositions when compared to L2TC patients: overall (198 vs 145 minutes [min], *P* < 0.001); discharged home (286 vs 160 min *P* < 0.001); non-ICU admissions (234 vs 164 min, *P* < 0.001), ICU admissions (123 vs 108 min, *P* < 0.001), and direct transport to the OR (126 vs 101 min, *P* < 0.001) (Table 3).

**Table 3.** Disposition of adult trauma patients treated at a level I vs level II trauma center.

Characteristic	Level I (n = 1,318,497)	Level II (n = 906,570)	*P*-value
Disposition from ED, n (%)			
Admit to floor	731,039 (55.4%)	517,613 (57.1%)	<0.001
Admit to ICU	254,892 (19.3%)	159,987 (17.6%)	<0.001
Direct to OR	180,479 (13.7%)	95,952 (10.6%)	<0.001
Discharged home	103,779 (7.9%)	88,399 (9.8%)	<0.001
ED LOS, minutes, median (IQR)			
All patients	198 (233)	145 (138)	<0.001
Admit to floor	234 (230)	164 (140)	<0.001
Admit to ICU	123 (163)	108 (108)	<0.001
Direct to OR	126 (196)	101 (117)	<0.001
Discharged home	286 (283)	160 (139)	<0.001

*ED*, emergency department; *ICU*, intensive care unit; *IQR*, interquartile range; *LOS*, length of stay; *OR*, operating room.

### Other Measured Outcomes of Level I- and II-Trauma Centers

When compared with L2TCs, the occurrence of an inhospital complication was higher at L1TCs (5.8% vs 4.4%, *P* < 0.001). This included increased rates of unplanned intubation (1.1% vs 0.8%, *P* < 0.001), ventilator- acquired pneumonia (0.5% vs 0.3%, *P* < 0.001), and deep vein thrombosis (0.7% vs 0.5%, *P* < 0.001) at L1TCs. Increased rates of unplanned ICU admissions (1.6% vs 1.3%, *P* < 0.001) and unplanned returns to the OR (0.7% vs 0.5%, *P* < 0.001) also occurred more commonly at L1TCs (Table 4). After adjusting for confounders, L1TC patients continued to exhibit a higher associated risk of complications (odds ratio [OR] 1.22, confidence interval [CI] 1.20–1.24, *P* < 0.001). Compared with L2TC patients, L1TC patients exhibited a higher rate of mortality (4.8% vs 3.8%, *P* < 0.001) (Table 4). However, this trend did not persist when controlling for known risk factors of mortality (OR 0.99, CI 0.97–1.01, *P* = 0.09).

**Table 4.** Outcomes for adult trauma patients treated at level I vs level II trauma centers.

Characteristic, n (%)	Level I (n = 1,318,497)	Level II (n = 906,570)	*P*-value
Any complication	76,217 (5.8%)	39,600 (4.4%)	<0.001
Cardiac arrest	12,623 (1.0%)	6,782 (0.7%)	<0.001
Catheter-associated UTI	2,329 (0.2 %)	1,073 (0.1%)	<0.001
Deep SSI	1,863 (0.1%)	646 (0.1%)	<0.001
Organ space SSI	1,432 (0.1%)	392 (<0.1%)	<0.001
Superficial SSI	1,360 (0.1%)	635 (0.1%)	<0.001
Deep vein thrombosis	8,802 (0.7%)	4,647 (0.5%)	<0.001
Pulmonary embolism	4,890 (0.4%)	2,016 (0.2%)	<0.001
Unplanned intubation	13,927 (1.1%)	7,400 (0.8%)	<0.001
Acute kidney injury	7,296 (0.6%)	3,971 (0.4%)	<0.001
Pressure ulcer	6,036 (0.5%)	2,948 (0.3%)	<0.001
Acute respiratory distress syndrome	3,884 (0.3%)	1,822 (0.2%)	<0.001
Unplanned return to OR	8,784 (0.7%)	4,126 (0.5%)	<0.001
Sepsis	4,107 (0.3%)	1,968 (0.2%)	<0.001
Stroke	3,581 (0.3%)	1,931 (0.2%)	<0.001
Unplanned ICU admission	21,417 (1.6%)	11,540 (1.3%)	<0.001
Ventilator-associated PNA	7,027 (0.5%)	2,654 (0.3%)	<0.001
Mortality rate, n (%)	63,347 (4.8%)	34,067 (3.8%)	<0.001

*ICU*, intensive care unit; *OR*, operating room; *PNA*, pneumonia; *SSI*, surgical site infection; *UTI*, urinary tract infection.

## DISCUSSION

This comprehensive five-year retrospective national analysis revealed that despite comparable injury burdens, patients treated at L1TCs experienced a longer associated ED-LOS across all disposition categories, along with a higher rate and associated risk of complications, compared to those at L2TCs. Interestingly, the associated risk of mortality remained similar between the two levels of trauma center designations.

Emergency department crowding remains a prominent issue, representing a pervasive challenge associated with delayed treatment, reduced patient satisfaction, and increased mortality.^32^ ^,^ ^33^ This situation occurs when the demand for emergency care surpasses the available resources in the ED, hospital, or both.^34^ Despite variations in definitions and measurements of crowding among hospitals, its repercussions will almost always result in a longer ED-LOS.^35^ White et al’s study, focusing on discharged patients, corroborates this correlation by revealing a 10% increase in ED-LOS for patients seen during periods of ED crowding.^36^ Crowding often leads to a bottleneck effect in patient flow, where patients awaiting admission occupy ED beds, thus limiting the availability for new patients. This scenario is further exacerbated during peak times or public health crises, like the COVID-19 pandemic, where an influx of patients can overwhelm ED resources. Prolonged wait times can lead to patient discomfort and dissatisfaction.^37^ Pines et al observed that extended ED-LOS was linked to a diminished probability of patients recommending the hospital to others, coupled with a lower perception of effective teamwork among hospital staff.^38^

The dynamics of resource utilization at trauma centers requires further investigation to uncover the underlying reasons for the observed prolonged ED-LOS at L1TCs. For instance, these centers are widely acknowledged for managing high patient volumes and catering to more complex cases that might hinder the flow of patients through the ED.^39^ This is supported by our study demonstrating that L1TCs more often treat patients with severe injuries to the head, abdomen, and thorax, compared to L2TCs. This may necessitate more comprehensive diagnostic evaluations, specialized interventions, and/or coordination among various surgical specialties, all of which contribute to longer LOS in the ED. Additionally, L1TCs often host residency programs and frequently involve residents and house staff in patient care, a feature less commonly found at L2TCs.^40^ The involvement of trainees may contribute to a longer ED-LOS, as residents and house staff may need to consult with attending physicians and supervisors to discuss treatment plans, which may lead to increased deliberation and decision-making time.^41^ ^–^ ^43^ Understanding the impact of educational programs on ED-LOS is necessary for optimizing resource allocation and enhancing efficiency of trauma care delivery within different levels of resource centers.

Patients at L1TCs were also more frequently impacted by social determinants of health including houselessness and substance use disorder. Our study revealed that L1TCs more often cared for houseless patients and those suffering from substance use disorder. Unhoused patients tend to experience longer ED-LOS since disposition planning and arrangements prove to be more complicated for patients lacking stable housing while their medical needs are being addressed.^44^ ^–^ ^46^ Moreover, houseless patients face markedly higher odds of hospital admission compared to their housed counterparts, a disparity likely influenced by clinicians’ concerns over the risks and safety of discharging individuals back to the streets.^47^ ^,^ ^48^ The pronounced presence of social determinants of health among patients at L1TCs highlights the complex interplay between healthcare delivery and societal issues, emphasizing the need for further investigation into healthcare disparities.

Increased ED-LOS may result in worsened clinical outcomes. We did not find a higher risk of mortality for patients treated at L1TCs; however, we did find a higher associated risk of inhospital complications. This pattern suggests suboptimal utilization or availability of important resources, potentially leading to the decompensation of patients. In support of this hypothesis, we found that patients at L1TCs had higher rates of unplanned intubation, ICU admission, and return to the OR. While the TQIP database is not granular enough to determine whether these complications were the result of increased ED-LOS, it does highlight the need for enhanced management strategies to ensure that patients receive timely and effective care, particularly in high-acuity settings where the margin for error is minimal.

Efficiently addressing the challenge of ED-LOS involves a multifaceted approach, integrating both strategic capacity management and innovative patient care practices. Key strategies include optimizing inpatient bed use, expanding ED capacity through additional beds or staffing, and early physician assessments to expedite decision-making, thereby reducing ED crowding and prolonged ED-LOS.^49^ Proven interventions such as the fast-track process, which notably reduced ED-LOS for low-acuity patients by 25%, and revised triage approaches in L1TCs have demonstrated success in expediting care and reducing ED-LOS.^50^ Another example of effective triage-system redesign involves establishing specialized units specifically for less severe cases, along with the inclusion of advanced practice practitioners. This approach has successfully led to a reduction in ED-LOS by more than 30 minutes.^51^

## LIMITATIONS

This study is limited by potential reporting and selection biases, coding errors, and missing data inherent in database studies. We did not consider a wide array of external factors that could influence ED-LOS, such as fluctuations in ED volume per center, disproportionate increases in centers approved, and variations in hospital and ED occupancy. Additionally, our study contains constraints in identifying specific treatment locations within the hospital, whether that be a dedicated trauma area or the general ED. A further limitation is our inability to control for competing LOS factors. Specifically, we were unable to account for factors including resident staffing, consult management, and the differing practice patterns for emergent and non-emergent care between the ED and other hospital settings.

Geographical differences between trauma centers were not considered, which might impact ED-LOS due to variations in regional healthcare policies, patient demographics, and resource availability. The TQIP database does not provide granular details on specific interventions and decision-making processes in the ED, which could affect LOS. Furthermore, we did not include patient socioeconomic factors in the analysis, which could have impacted ED-LOS and patient outcomes. Finally, as with all database studies, we cannot conclude any definitive causality statement regarding trauma center level and ED-LOS. Despite these limitations, our findings contribute significantly to the discourse on ED-LOS, laying a foundation for future research aimed at optimizing resource allocation and improving trauma care delivery in L1TCs and L2TCs.

## CONCLUSION

This comprehensive analysis highlights a significant observed disparity in ED length of stay between Level I and Level II trauma centers. Level I trauma centers consistently reported longer associated ED-LOS across various patient dispositions, as well as a higher risk of complications, despite treating similarly injured patients. Factors leading to these findings could range from operational protocols and resource management to patient case complexity and institutional policies. Due to limitations of the Trauma Quality Improvement Program database, we were unable to attribute the observed differences in ED-LOS to any single factor, as the associations observed in our study are based on data from a large national database, which enhances the generalizability of our findings across diverse settings. This broad scope reduces the influence of regional policies and allows our results to be applicable on a wider scale. However, addressing these underlying causes is essential not only for enhancing the efficiency of patient flow through the hospital but also for improving the overall quality of care provided to trauma patients. To effectively tackle this issue, further prospective research is needed to delve into the specifics of why these discrepancies exist. This includes examining hospital operational strategies, patient flow processes, staffing models, and the use of technology in patient management.
